# High-Breakdown and Low-Leakage 4H-SiC MOS Capacitor Based on HfO_2_/SiO_2_ Stacked Gate Dielectric in Trench Structures

**DOI:** 10.3390/nano15050343

**Published:** 2025-02-22

**Authors:** Qimin Huang, Yunduo Guo, Anfeng Wang, Lin Gu, Zhenyu Wang, Chengxi Ding, Yi Shen, Hongping Ma, Qingchun Zhang

**Affiliations:** 1 Institute of Wide Bandgap Semiconductors and Future Lighting, Academy for Engineering & Technology, Fudan University, Shanghai 200433, China22210860078@m.fudan.edu.cn (A.W.); qingchun_zhang@fudan.edu.cn (Q.Z.); 2 Shanghai Research Center for Silicon Carbide Power Devices Engineering & Technology, Fudan University, Shanghai 200433, China; 3 Institute of Wide Bandgap Semiconductor Materials and Devices, Research Institute of Fudan University in Ningbo, Ningbo 315327, China

**Keywords:** trench MOS, ALD, high-k, gate dielectric, MIS capacitor

## Abstract

The progression of SiC MOSFET technology from planar to trench structures requires optimized gate oxide layers within the trench to enhance device performance. In this study, we investigated the interface characteristics of HfO_2_ and SiO_2_/HfO_2_ gate dielectrics grown by atomic layer deposition (ALD) on SiC trench structures. The trench structure morphology was revealed using scanning electron microscopy (SEM). Atomic force microscopy (AFM) measurements showed that the roughness of both films was below 1nm. Spectroscopic ellipsometry (SE) indicated that the physical thicknesses of HfO_2_ and SiO_2_/HfO_2_ were 38.275 nm and 40.51 nm, respectively, demonstrating their comparable thicknesses. X-ray photoelectron spectroscopy (XPS) analysis of the gate dielectrics revealed almost identical Hf 4f core levels for both HfO_2_ and the SiO_2_/HfO_2_ composite dielectrics, suggesting that the SiO_2_ interlayer and the SiC substrate had minimal impact on the electronic structure of the HfO_2_ film. The breakdown electric field of the HfO_2_ film was recorded as 4.1 MV/cm, with a leakage current at breakdown of 1.1 × 10^−3^A/cm^2^. The SiO_2_/HfO_2_ stacked film exhibited significantly better performance, with a breakdown electric field of 6.5 MV/cm and a marked reduction in leakage current to 3.7 × 10^−4^ A/cm^2^. A detailed extraction and analysis of the leakage current mechanisms were proposed, and the data suggested that the introduction of thin SiO_2_ interfacial layers effectively mitigated small bandgap offset issues, significantly reducing leakage current and improving device performance.

## 1. Introduction

SiC MOSFETs offer significant advantages over traditional silicon materials owing to their excellent performance in terms of high-temperature, high-frequency, and high breakdown voltage, wider bandgap, better thermal conductivity, and stronger radiation resistance [1]. These properties make SiC MOSFETs suitable for use in various application fields, including power electronics, electric vehicles, and renewable energy conversion systems. However, transitioning from planar to trench structures in SiC MOSFETs significantly enhances current-carrying capability, reduces on-resistance, increases switching speed, and enables higher rated voltages [2,3]. However, the introduction of trench structures is still challenging for the gate oxide layers [4,5], due to the much higher critical breakdown field strength of SiC than that of traditional materials, as well as the high electric field concentration at the gate corners in trench structures resulting in severe stress on the gate oxide layer [6,7]. Therefore, optimizing the gate oxide layer and solving interface engineering issues have become key research priorities in materials science and engineering. For instance, Wirths et al. [8,9] fabricated planar SiC MOSFETs by incorporating high dielectric constant (high-k) gate dielectrics as a replacement for conventional SiO_2_ and the yield significantly improved device performance in terms of reduced interface trap density, enhanced threshold voltage stability, and improved device reliability [10,11,12]. Consequently, high-k materials offer a promising approach to enhancing gate oxide performance.

HfO_2_ with a high dielectric constant (k = 20) and wide bandgap (5.8 eV) is a promising gate dielectric material [13]. However, employing HfO_2_ as the gate dielectric in 4H-SiC-based MOS devices may lead to high leakage currents due to the low conduction band offset between HfO_2_ and 4H-SiC [14], which prevents the dielectric from effectively blocking leakage current [15]. Wang et al. conducted first-principles calculations and suggested that introducing a thin SiO_2_ layer between HfO_2_ and the SiC substrate could effectively mitigate this issue [16]. Building on this, we implemented this stacked structure in SiC trenches for further investigation. This approach may enhance the band offset, suppress leakage current, and simultaneously preserve the advantages of HfO_2_’s high dielectric constant [17]. On the other hand, traditional thermal oxidation processes induce an abnormally high interface trap density (Dit) at the SiC/SiO_2_ interface, which can severely degrade channel mobility [18,19].

To solve the above-considered issues, atomic layer deposition (ALD) was employed in this work to form uniform and high-quality gate oxide layers [20,21]. This deposition method enables precise control over film thickness, composition, and uniformity while facilitating the atomic-scale regulation of the deposition process. Consequently, ALD serves as a powerful technique for studying the microscopic growth process, growth mechanisms, and defect formation in thin films. The impact of the interface was investigated by fabricating HfO_2_ and SiO_2_/HfO_2_ gate dielectrics in trench capacitors. The depth and width of the trench structure and the growth of the high-k films were viewed by SEM. The growth of high-quality films was further confirmed by AFM, spectroscopic ellipsometry (SE), and X-ray photoelectron spectroscopy (XPS). The film thickness was measured using SE, and the bandgap was calculated. It was found that the addition of the SiO_2_ layer affected the bandgap. The device breakdown electric field (*E*_BR_) and leakage current at the onset of breakdown (*I*_ON_EBR_) were obtained through current-voltage (I–V) measurements along with fitting analysis of the leakage current mechanisms. Capacitance-voltage (C–V) measurements were performed to obtain C–V hysteresis curves, from which the interface trap density (*D*_it_) and effective oxide charge (*N*_eff_) were calculated. Fitting analysis of I–V characteristics revealed the different leakage current mechanisms for the two gate dielectrics. This study found that the SiO_2_/HfO_2_ stacked dielectric exhibited higher breakdown voltage and lower leakage current, demonstrating feasibility and potential as a gate dielectric in trench structures.

## 2. Experimental Section

**Figure 1:**
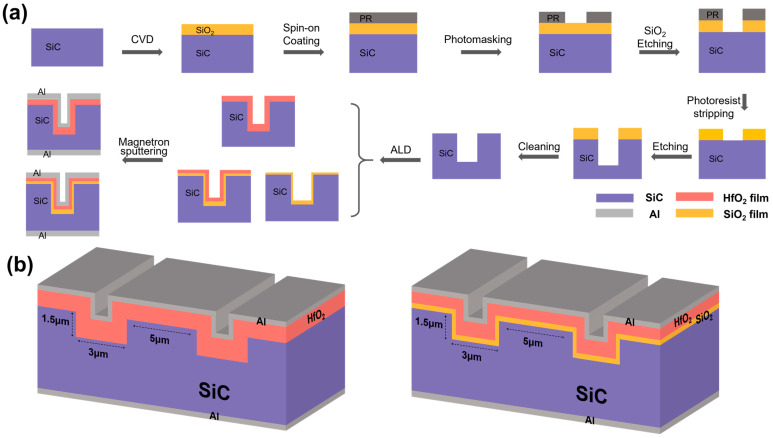
(**a**) Schematic diagram of manufacturing process flow for trench MOS and (**b**) scheme of the final fabricated sample structure.

The sample preparation process is shown in Figure 1a. Firstly, a uniform SiO_2_ film was deposited on the surface of an n-type 4° off-angle silicon face (0001) 4H-SiC epitaxial wafer by chemical vapor deposition (CVD) to form a masking layer. Following the application of a photoresist, photolithography was used to pattern the SiO_2_ masking layer and define the target trench structure. The photolithography process involved exposure and development processes to create precise patterns on the mask layer, followed by controlling subsequent etching steps to ensure accurate trench dimensions and shapes. The trench structure was aligned along the $1\bar{1}00$ crystallographic phase of the SiC substrate. After etching the SiO_2_ masking layer, the photoresist was stripped, and the SiC etching was performed to form the desired trench structure. Next, buffered oxide etch (BOE) technology was used to remove the unprotected areas of the masking layer to form the trench structure. The obtained trench possessed a width of 3 µm, a depth of 1.5 µm, and a spacing of 5 µm, providing a precise structural base for the subsequent thin film deposition. After completion of the trench structure, ALD was employed to uniformly deposit an approximately 40 nm thick gate oxide dielectric layer onto the trench surface. The deposition was performed for 400 cycles at a temperature of 270 °C. TDMAHf and H_2_O were used as the precursors for Hf and O, respectively, to deposit HfO_2_ films. In the deposition of SiO_2_, Bis(diethylamino)silane (BDEAS) was served as the Si precursor, while plasma was used as the O source. The use of ALD technology ensured precise control over the thickness of each layer and guaranteed the formation of a uniform and stable gate oxide layer. Finally, magnetron sputtering was utilized to deposit a 200 nm thick aluminum electrode layer on both the top and bottom sides of the sample. To ensure good electrical performance and stable electrical contact, the front shape of the aluminum electrode was designed as a square with dimensions of 200 µm × 200 µm. The schematic diagrams of the final HfO_2_ thin film and SiO_2_/HfO_2_ bilayer thin film samples are depicted in Figure 1b. This device includes the coexistence of top and trench sidewall dielectric interfaces.

**Figure 2:**
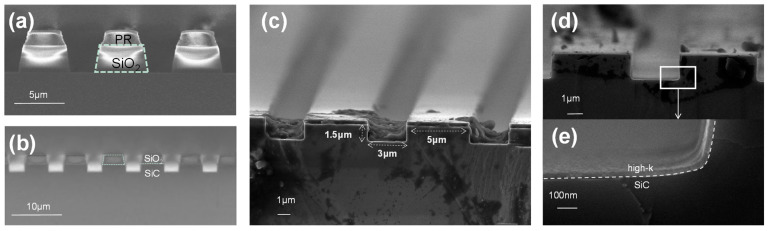
SEM images (**a**) after SiO_2_ etching, (**b**) after SiC etching, (**c**) of the trench structure after BOE cleaning, (**d**) after ALD processing, and (**e**) of magnification of the white-boxed area in (**d**).

During the manufacturing process, the trench structures at different stages were observed by SEM technique and images of trench structure after etching the SiO_2_ and SiC layers are presented in Figure 2a,b. The mask layer was then removed by using BOE to yield a satisfactory trench structure with a width of 3 μm, depth of 1.5 μm, and spacing of 5 μm (Figure 2c). As depicted in the SEM image of Figure 2d, the grown gate oxide layer after ALD exhibited excellent consistency even at the trench corners (Figure 2e), highlighting the advantages of ALD when compared to traditional dry oxidation methods in terms of better control over film thickness and uniformity.

The performance of the fabricated devices was evaluated through a series of tests. After the etching process and ALD treatment, the samples were cleaved, and their backsides were attached to a cross-sectional stage. Following a 30 s gold sputtering process, cross-sectional imaging was performed using a Sigma 300 SEM (ZEISS, Oberkochen, Germany) to assess trench etching and film growth. Additional material tests and analyses were then performed on the films after ALD. The film thickness was measured by SE (HORIBA France SAS, Palaiseau, France) equipment. The SiC substrate was parameterized using B-splines, and the thickness and associated optical parameters of the films were determined using the Cauchy dispersion model to characterize the gate dielectric layer. Surface topography analysis was performed using atomic force microscopy (AFM) (Bruker BRK0003, Karlsruhe, Germany) with a scanning range of 25 µm^2^. The raw data were processed using NanoScopeAnalysis (3.0) (Billerica, MA, USA) software to determine surface roughness. Further analysis of the chemical states of the films was carried out by XPS (Thermo ScientificTM ESCALAB 250Xi, Waltham, MA, USA) spectrometry. To ensure accurate data, a 10 nm etching was performed on the surfaces of both films. All spectral peaks were calibrated using a C 1s peak with a binding energy of 284.8 eV.

The fabricated devices were tested by a TS2000-HP probe station (Hsinchu, Taiwan) and a Keithley 4200A-S (Solon, OH, USA) semiconductor analyzer. The I–V curves were obtained through a gradual increase in gate voltage until reaching dielectric breakdown. The *E*_BR_ and *I*_ON_EBR_ of MOS samples were determined from the I–V curves. The C–V curves at different frequencies were obtained by scanning the gate voltage at an AC voltage of 30mV. *D*_it_ and *N*_eff_ values of the MOS capacitors’ oxide layer were calculated from the C–V data and all tests were conducted at room temperature.

## 3. Results and Discussion

**Figure 3:**
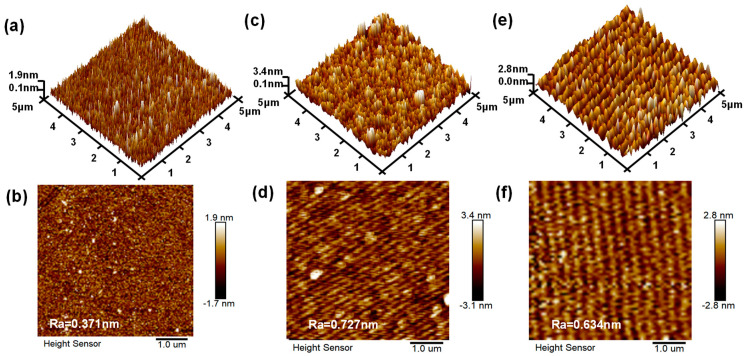
Both 3D and 2D AFM images within the area of 5 × 5 um^2^ for (**a**,**b**) HfO_2_ and (**c**,**d**) SiO_2_/HfO_2_, and (**e**,**f**) interlayer SiO_2_.

The surface morphologies and roughnesses of HfO_2_ and SiO_2_/HfO_2_ films samples and the interlayer SiO_2_ were characterized by AFM and the results are compared in Figure 3. The arithmetic mean roughness (*R*_a_) was calculated based on a measured area of 5 × 5 µm^2^. Both surfaces remained relatively flat, with a determined *R*_a_ of 0.371 nm for HfO_2_ film and 0.727 nm for SiO_2_/HfO_2_ stacked film. Hence, the morphology and uniformity of films prepared by the ALD technique can be well controlled. However, the SiO_2_/HfO_2_ film exhibited comparatively higher surface roughness than HfO_2_, attributed to the different physical and chemical properties of SiO_2_ and HfO_2_ that may induce interfacial stress and defects during deposition to yield increased overall surface roughness. Furthermore, the multilayer structure may contribute since each added layer in the SiO_2_/HfO_2_ stack could introduce cumulative minor roughnesses, resulting in higher final surface roughness than the single HfO_2_ layer. In the SiO_2_/HfO_2_ stack structure, the presence of the SiO_2_ layer may introduce a slight increase in interface roughness. However, the surface roughnesses of both the HfO_2_ film and the SiO_2_/HfO_2_ stack remained at a low level overall.

**Figure 4:**
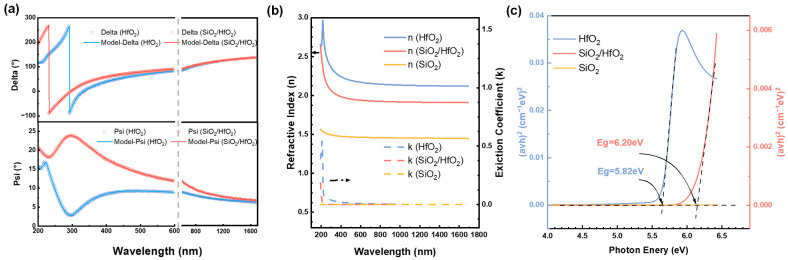
SE results for HfO_2_ and SiO_2_/HfO_2_ samples: (**a**) *Ψ* (psi) and *Δ* (delta) of the measured complex reflectance, (**b**) fitted refractive index and extinction coefficient, and (**c**) (*αhν*)^2^ parameter versus photon energy for the fitted bandgap.

A total of five point measurements were performed by SE at various locations of the films, and the mean values were calculated and used for analysis. The thickness of the HfO_2_ film was determined as 38.257 nm, while that of SiO_2_ and HfO_2_ layers in the SiO_2_/HfO_2_ composite film was estimated to be 10.73 nm and 29.78 nm, respectively. The measured psi(Ψ) and delta(Δ) curves are presented in Figure 4a, and the refractive index (*n*) and extinction coefficient (*k*) are given in Figure 4b. The absorption coefficient (α) was calculated by the following formula: α = 4π*k*/*λ* [22], where k is the extinction coefficient presented as a function of wavelength (*λ*), as shown in Figure 4c. The Tauc method was subsequently employed to plot the relationship between (*α*h*υ*)^2^ and photon energy (h*υ*) for extracting the optical bandgap through linear extrapolation [23]. From Figure 4, the optical bandgaps of HfO_2_ film and SiO_2_/HfO_2_ composite film were calculated as 5.82 eV and 6.20 eV, respectively [24]. Despite the low thickness of the introduced SiO_2_ film, its high bandgap width (approximately 9 eV) [25] allowed effective bandgap adjustment, conducive to breakdown voltage enhancement and leakage current reduction in the devices.

**Figure 5:**
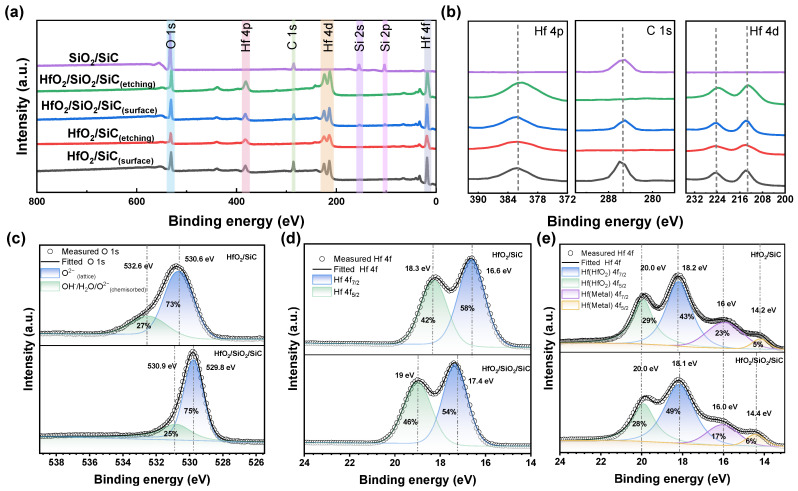
(**a**) XPS spectra of HfO_2_ and SiO_2_/HfO_2_ sample surfaces and the etched and interlayer SiO_2_, (**b**) O 1s core level, (**c**) sample surface, (**d**) etched Hf 4f core level, and (**e**) magnified view of Hf 4p, C 1s, and Hf 4d peaks.

The elemental compositions and chemical states of the film surfaces were thoroughly investigated by XPS analysis. Figure 5a,b present the wide XPS spectra of HfO_2_ and SiO_2_/HfO_2_ dielectric surfaces before and after etching, as well as detailed spectra of the Hf 4p, C 1s, and Hf 4d peaks, including analysis of the SiO_2_ interlayer. The spectra displayed characteristic electron peaks of HfO_2_ corresponding to Hf 4f, Hf 4d, and Hf 4p [26], along with the XPS peaks of O 1s and C 1s. The comparison of HfO_2_ and SiO_2_/HfO_2_ films before and after etching revealed an increase in C 1s peak before etching, indicative of slight surface contamination. As shown in Figure 5b, the Hf 4d and Hf 4p peaks of both samples were identical.

The O 1s core-level XPS spectra and peak deconvolution of the HfO_2_ and SiO_2_/HfO_2_ films are illustrated in Figure 5c. The O 1s peak of the HfO_2_ film was divided into two peaks at 530.6 eV and 531.4 eV, while the O 1s peak of the SiO_2_/HfO_2_ film was split into two peaks at 529.4 eV and 530.5 eV. The Hf 4f core-level XPS spectra and peak separations of the surfaces of HfO_2_ and SiO_2_/HfO_2_ films in Figure 5d revealed well-separated Hf 4f_7/2_ and Hf 4f_5/2_ peaks by approximately 1.7 eV and 1.6 eV [27], respectively. The Hf 4f core-level XPS spectra and peak separations within the HfO_2_ and SiO_2_/HfO_2_ films are provided in Figure 5e. Besides the separation of the Hf 4f_7/2_ and Hf 4f_5/2_ peaks in HfO_2_, metallic Hf 4f_7/2_ and Hf 4f_5/2_ peaks were also observed [28], which may result from the reduction of HfO_2_ by high-energy ions during the etching process, forming metallic Hf. In the HfO_2_ film, the binding energies of the Hf 4f_7/2_ and Hf 4f_5/2_ peaks were estimated to be 18.2 eV and 20.0 eV, respectively. The area ratio of the Hf 4f_7/2_ to Hf 4f_5/2_ peaks in the HfO_2_ film was recorded as 43:29, while that of SiO_2_/HfO_2_ film was 49:28, with both values equivalent to a ratio of approximately 4:3 [28]. The latter would correspond to the occupancy levels caused by spin-orbit coupling, primarily determined by degeneracy.

The results of XPS indicated almost identical Hf 4f core levels in HfO_2_ and SiO_2_/HfO_2_ composite dielectrics, suggesting the negligible impact of the SiO_2_ interlayer and the SiC substrate on the electronic structure of the HfO_2_ film. This can be related to the characteristics of the ALD process, ensuring high uniformity and stability during the formation of interfacial layers for minimized interface effects. Furthermore, the previously mentioned energy bandgap (*E_g_*) widening observed in the stacked samples is likely due to the presence of SiO_2_.

**Figure 6:**
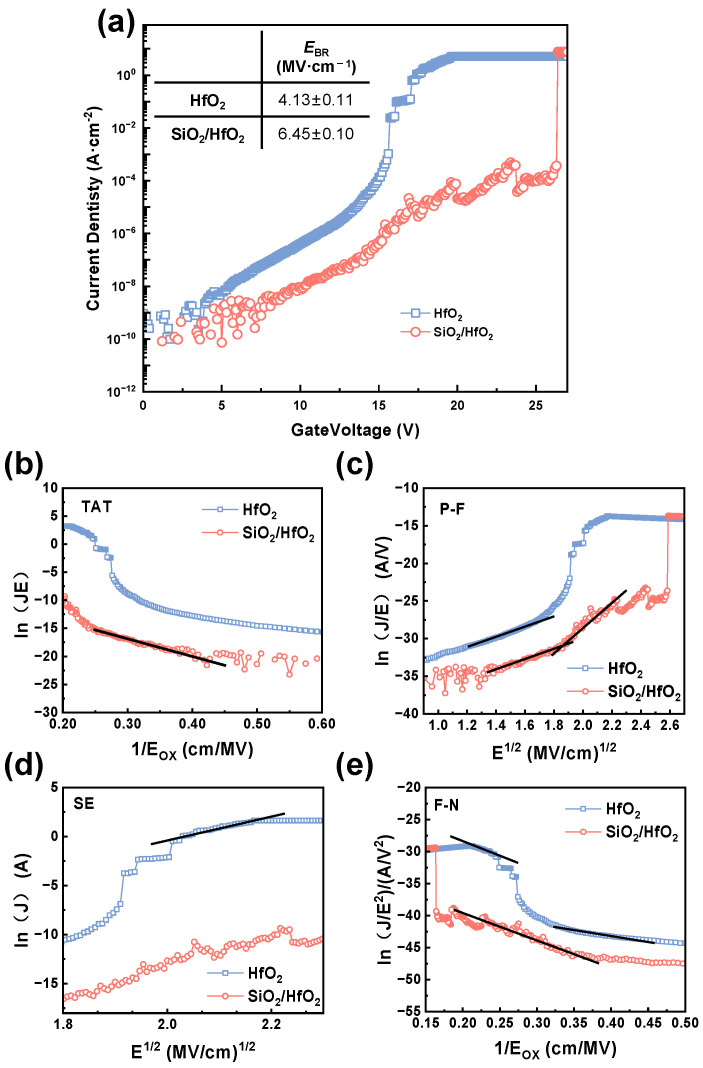
(**a**) Current density (*J*)-voltage (*V*) curves of MOS capacitors. Electric fields of HfO_2_ and SiO_2_/HfO_2_ dielectric MOS capacitors for (**b**) TAT, (**c**) Frenkel–Poole emission, (**d**) Schottky emission, and (**e**) F–N tunneling model fitting plot.

The electrical properties of the dielectrics were examined by testing the fabricated MOS capacitors with a probe station and a semiconductor analyzer. The current density (*J*)–voltage (*V*) characteristics of HfO_2_ and SiO_2_/HfO_2_ dielectric MOS capacitors are compared in Figure 6a, where *J* is normalized to the area of the square electrode and calculated by the following formula: $J=\frac{I}{A}$, with I representing the measured current and *A* donating the electrode area [29]. As the electric field increased, the single-layer HfO_2_ capacitor exhibited a sudden jump in leakage current but without a significant breakdown phenomenon. After the breakdown, the current continued to rise, indicating a soft breakdown state. The breakdown voltage was estimated to be approximately 15.6 V, corresponding to a breakdown field of 4.1 MV/cm. For the stacked SiO_2_/HfO_2_ sample, the breakdown field at 26.3 V was calculated as 6.5 MV/cm, indicating a slightly improved breakdown field after the addition of the wide-bandgap dielectric SiO_2_ at the HfO_2_/SiC interface. The mean and standard deviation of the breakdown field for five sets of samples were calculated, as shown in the table in Figure 6a. For the single-layer HfO_2_, *I*_ON_EBR_ was determined as 1.1 × 10^−3^ A/cm^2^, whereas that of stacked SiO_2_/HfO_2_ was significantly lower (3.7 × 10^−4^ A/cm^2^). The leakage current and breakdown voltage increased after adding the SiO_2_ layer.

The conduction mechanisms under low leakage conditions were further analyzed using current density (J) – electric field (E) data extracted from experimental characteristics and used to construct conduction mechanism plots for different gate electric field regions [30,31]. Model parameters were extracted from the linear features of these plots and applied to simulate the analytical model equations for each conduction mechanism.

**Figure 7:**
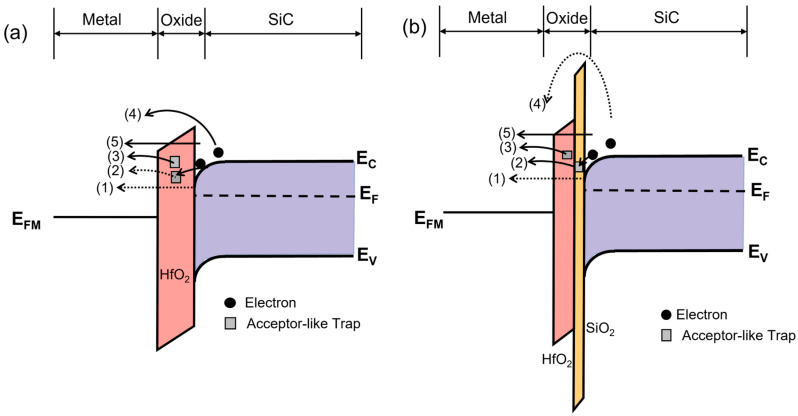
Schematic energy-band diagrams of (**a**) HfO_2_ and (**b**) SiO_2_/HfO_2_ MOS structures. The numbering 1–5 refers to respective leakage paths due to direct tunneling, trap-assisted tunneling, Poole–Frenkel emission, Schottky emission, and Fowler–Nordheim tunneling. *E_C_*, *E_F_*, *E_V_*, and *E_FM_* are the conduction band edge, Fermi level, valence band edge, and Fermi level of Al, respectively.

The gate leakage current in MOS capacitors would arise from various mechanisms, including direct tunneling (DT, path 1 in Figure 7), trap-assisted tunneling (TAT), Poole–Frenkel (P–F) emission, Schottky emission, and Fowler–Nordheim (F–N) tunneling. Since direct tunneling would typically occur in ultrathin dielectric layers, more focus was paid to TAT, P–F emission, Schottky emission, and F–N tunneling. The fitting results for different mechanisms based on the extracted parameters are presented in Figure 6b–e, and the model parameters and analytical equations for the mechanisms are listed in Table 1.

Trap-assisted tunneling (TAT), according to Path 2 in Figure 7, occurred when electrons first tunneled into traps within the dielectric thin film and then into the conduction band of the dielectric [32]. In Figure 6b, HfO_2_ displayed a lower trap density, resulting in a minimal contribution from the TAT mechanism. By contrast, the SiO_2_/HfO_2_ stack exhibited significant TAT activity due to the high-density traps introduced by the interface SiO_2_ layer. These traps captured and released electrons under low voltage, forming the TAT pathways.

Field-assisted thermal de-trapping of carriers from traps in the dielectric bulk into the conduction band resulted in P-F emission (path 3 in Figure 7) [34]. In Figure 3c, P–F emission primarily occurred under medium to high electric fields. Schottky emission (Path 4 in Figure 7) described the transition of electrons from a semiconductor into the conduction band of the insulating layer [34], a process analogous to thermionic emission in metal–semiconductor junctions. When the voltage rose to the medium field range, the P–F emission mechanism became active in the HfO_2_ film, releasing some electrons from the traps. In Figure 6d, the low barrier height simultaneously promoted the occurrence of Schottky emission. In the SiO_2_/HfO_2_ stack, the PF emission mechanism also became prominent and worked synergistically with TAT. However, the high barrier of the SiO_2_ layer suppressed Schottky emission, preventing its occurrence in this structure.

At higher gate voltages, electrons started to tunnel through a triangular potential barrier, resulting in F–N tunneling under high electric fields (Path 5 in Figure 7) [35]. As illustrated in Figure 6e, F–N tunneling dominated the current conduction in the HfO_2_ film at high fields, causing a rapid rise in current density. For the SiO_2_/HfO_2_ stack, the introduction of SiO_2_ significantly enhanced the barrier height, shifting the onset of F–N tunneling to higher electric fields. As a consequence, the increase in current was delayed when compared to pure HfO_2_ films. Additionally, even after the occurrence of hard breakdown in the HfO_2_ film, a portion of F–N tunneling persisted.

In sum, leakage current in the HfO_2_ film was primarily driven by P–F emission, Schottky emission, and F–N tunneling, while that of the SiO_2_/HfO_2_ stack was predominantly contributed by TAT, P–F emission, and F–N tunneling mechanisms.

**Figure 8:**
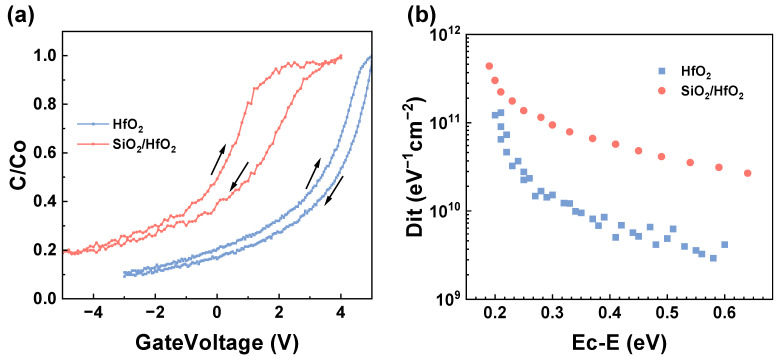
(**a**) Normalized C-V hysteresis curves of MOS capacitors with HfO_2_ and SiO_2_/HfO_2_ dielectrics, and (**b**) calculated interface trap density *D*_it_ plots.

The interface characteristics were examined by obtaining normalized C–V hysteresis curves of HfO_2_ and SiO_2_/HfO_2_ dielectric MOS capacitors measured at 1 MHz and the data are summarized in Figure 8a. Notably, the measurements were conducted with a 30 mV AC signal swept from negative to positive voltages and then returned from high to low voltages. The flat band voltage (*V*_FB_) was determined from the normalized capacitance of the MOS. The first step in this process consisted of calculating the flat band capacitance (*C_FB_*) using the following equation [36,37]:

(1) $$\frac{C_{FB}}{C_{ox}}=\frac{1}{1+\frac{\varepsilon_{ox}}{\varepsilon_{SiC}t_{ox}}\sqrt{\frac{\varepsilon_{0}\varepsilon_{SiC}kT}{q^{2}n_{0}}}}$$

where *C_ox_* represents the oxide capacitance per unit area, *t_ox_* is the thickness of the oxide layer, *n_0_* denotes the majority carrier concentration in the epitaxial layer, and *ε_ox_* and *ε_SiC_* are the relative permittivities of the oxide and SiC, respectively. For the SiO_2_/HfO_2_ stacked structure, the weighted average method was employed to account for the permittivity and thickness of both materials ($\varepsilon_{\mathrm{ox}}=\frac{d_{{\mathrm{SiO}}_{2}}}{d_{\mathrm{total}}}\cdot \varepsilon_{{\mathrm{SiO}}_{2}}+\frac{d_{{\mathrm{HfO}}_{2}}}{d_{\mathrm{total}}}\cdot \varepsilon_{{\mathrm{HfO}}_{2}}$) [38].

The calculated dielectric constant of the SiO_2_/HfO_2_ stack was approximately 16. The *V*_FB_ values for the HfO_2_ and SiO_2_/HfO_2_ dielectric MOS capacitors were calculated as, respectively, 1.65 V and 0.8 V, showing significantly reduced flat-band voltage shift after the introduction of the SiO_2_ stack.

During high-frequency C–V testing, border traps cannot respond to small AC signal variations, but they can charge and discharge with the DC gate voltage sweep. Consequently, sweeping the gate voltage from negative to positive and from positive to negative during the test would result in hysteresis between the high-frequency dual C–V curves due to the presence of border trap charges. Since samples with stacked dielectrics exhibited larger hysteresis than the non-stacked HfO_2_ samples, it can be concluded that the HfO_2_ dielectric possessed relatively lower slow trap densities, which can be further demonstrated by calculating *D*_it_.

As depicted in Figure 8b, the *D*_it_ calculated using the high-low-frequency method indicated a *D*_it_ value of 1.30 × 10^11^ eV^−1^·cm^−2^ for HfO_2_ and 4.38 × 10^11^ eV^−1^·cm^−2^ for SiO_2_/HfO_2_ stack at *E_C_* −0.2 eV. The SiO_2_/HfO_2_ bilayer films exhibited relatively high overall interface trap density, inferring the presence of certain defects and consistent with the TAT mechanism observed under medium electric fields. The higher interface trap density may also be attributed to the increased surface roughness of the SiO_2_/HfO_2_ bilayer. Also, elevated *D*_it_ values may be linked to the presence of defects introduced during the etching process, and interface quality might be improved through annealing treatments. However, overall, the *D*_it_ values of both samples remained in the same order of magnitude with minimal fluctuation.

The effective oxide charge (*N*_eff_) value in the oxide layer can be calculated using *V*_FB_ [39,40]:

(2) $$N_{eff}=(\frac{W_{m}-W_{s}}{q}-V_{FB})\cdot C_{ox}$$

where *W_m_* denotes the work function of Al metal (4.28 eV) and *W_s_* is the work function of the semiconductor material.

The function of SiC can be calculated as follows:

(3) $$W_{s}=\chi +\frac{E_{g}}{2}-kTln(\frac{n_{0}}{n_{i}})$$

where *χ*, *E_g_*, and *n_i_* are the electron affinity, bandgap, and intrinsic carrier concentration of SiC, respectively. Notably, $\frac{W_{m}-W_{s}}{q}$ represents the ideal flat-band voltage without considering the charge in the oxide layer.

By substituting Equation (3) into Equation (2), the effective charge *N*_eff_ can be obtained. The *N*_eff_ values for HfO_2_ and SiO_2_/HfO_2_ dielectric MOS capacitors were recorded as −1.45 × 10^11^ cm^−2^ and −1.01 × 10^11^ cm^−2^, respectively. The electrical and interface characteristics of MOS capacitors based on HfO_2_ and SiO_2_/HfO_2_ dielectrics in Table 2 revealed negative *N*_eff_ to cause a forward shift in the *V*_FB_. The *N*_eff_ in the oxide layer consisted of *D*_it_, fixed charge, oxide-trapped charge, and mobile charge [41]. These charges significantly impacted the electrical characteristics of trench MOSFETs, such as threshold voltage, flat-band voltage, and channel mobility. Therefore, the SiO_2_/HfO_2_ stacked structure improved the device’s interface quality by introducing a SiO_2_ interlayer able to reduce *V*_FB_ shift and decrease the effective oxide charge.

## 4. Conclusions

In summary, ALD technology was successfully employed to grow HfO_2_ and SiO_2_/HfO_2_ gate dielectric films on etched 4H-SiC trench structures and fabricated corresponding MOS capacitors. The tests were conducted on a combined structure with both planar and vertical surfaces. AFM measurements showed that the roughness of both films was less than 1 nm, indicating relatively smooth surfaces. The test results showed HfO_2_ film exhibiting a breakdown electric field of 4.1 MV/cm with a leakage current of 1.1 × 10^−3^ A/cm^2^ at breakdown. By comparison, the SiO_2_/HfO_2_ film demonstrated a significantly higher breakdown electric field of 6.5 MV/cm and a much lower leakage current of 3.7 × 10^−4^ A/cm^2^. Leakage mechanism analysis revealed a leakage current in the HfO_2_ film, primarily driven by P–F emission, Schottky emission, and F–N tunneling, whereas the SiO_2_/HfO_2_ stack was dominated by TAT, P–F emission, and F–N tunneling. In the C-V hysteresis, a larger hysteresis was observed for the SiO_2_/HfO_2_ stacked dielectric, which is consistent with the increased roughness and *D*_it_ caused by the additional interface. However, the differences in roughness and *D*_it_ between the two samples were not significant, which can be attributed to the compact structure formed by the ALD process. The introduction of a thin SiO_2_ layer effectively solved the small bandgap offset, thereby increasing the bandgap from 5.82 eV to 6.20 eV and contributing to the reduction in leakage current. Furthermore, the etching process induced interface damage in the trench structure, potentially affecting device performance. Post-treatments could be considered to improve interface quality, which will be the focus of future research. Overall, the comprehensively analyzed performances of HfO_2_ and SiO_2_/HfO_2_ as gate dielectrics in 4H-SiC trench structures provide valuable references for future potential applications in 4H-SiC trench MOSFETs.

## Figures and Tables

**Table 1:** Conduction plots and analytical model equations of different gate leakage current mechanisms.

Conduction Mechanisms	Conduction Plot	Analytical Model Equations	
TAT	ln(JE) ∝ $\frac{1}{E}$	$J_{TAT}=\frac{2qc_{T}N_{T}\phi_{T}\exp\left(-\frac{D{\phi_{T}}^{\frac{3}{2}}}{E_{ox}}\right)}{3E_{ox}}$	[32]
P–F emission	ln($\frac{J}{E}$) ∝ $\sqrt{E}$	$J_{PF}=qN_{c}\mu E_{ox}\exp\left(\frac{-q\left(\phi_{t}-\sqrt{\frac{qE_{ox}}{\pi \varepsilon_{0}k_{r}}}\right)}{kT}\right)$	[18]
Schottky emission	ln(J) ∝ $\sqrt{E}$	$J_{SE}=A^{*}T^{2}\exp\left(\frac{-q\left(\phi_{B}-\sqrt{\frac{qE_{ox}}{4\prod \varepsilon_{ox}}}\right)}{kT}\right)$	[18]
F–N tunneling	ln($\frac{J}{E^{2}}$) ∝ $\frac{1}{E}$	$J_{FN}=A{E_{ox}}^{2}\exp\left(-\frac{B}{E_{ox}}\right)$	[33]

**Table 2:** Electrical and interface characteristics of MOS capacitors based on HfO_2_ and SiO_2_/HfO_2_ dielectrics.

Stack Information	*V*_FB_ (V)	*E*_BR_ (MV·cm^−1^)	*I*_ON_EBR_ (A·cm^−2^)	*D*_it_ (eV^−1^·cm^−2^) E_C_-0.2 eV	*N*_eff_ (cm^−2^)
HfO_2_	1.65	4.1	1.1 × 10^−3^	1.30 × 10^11^	−1.45 × 10^11^
SiO_2_/HfO_2_	0.8	6.5	3.7 × 10^−4^	4.38 × 10^11^	−1.01 × 10^11^

## Data Availability

The original contributions presented in this study are included in the article. Further inquiries can be directed to the corresponding author.
